# Robust Automated Harmonization of Heterogeneous Data Through Ensemble Machine Learning: Algorithm Development and Validation Study

**DOI:** 10.2196/54133

**Published:** 2025-01-22

**Authors:** Doris Yang, Doudou Zhou, Steven Cai, Ziming Gan, Michael Pencina, Paul Avillach, Tianxi Cai, Chuan Hong

**Affiliations:** 1 Department of Biomedical Informatics, Harvard Medical School, Boston, MA, United States; 2 Department of Statistics and Data Science, National University of Singapore, Singapore, Singapore; 3 Department of Computer Science, Rensselaer Polytechnic Institute, Rochester, NY, United States; 4 Department of Statistics, University of Chicago, Chicago, IL, United States; 5 Department of Biostatistics & Bioinformatics, Duke University, Durham, NC, United States

**Keywords:** ensemble learning, semantic learning, distribution learning, variable harmonization, machine learning, cardiovascular health study, intracohort comparison, intercohort comparison, gold standard labels

## Abstract

**Background:**

Cohort studies contain rich clinical data across large and diverse patient populations and are a common source of observational data for clinical research. Because large scale cohort studies are both time and resource intensive, one alternative is to harmonize data from existing cohorts through multicohort studies. However, given differences in variable encoding, accurate variable harmonization is difficult.

**Objective:**

We propose SONAR (Semantic and Distribution-Based Harmonization) as a method for harmonizing variables across cohort studies to facilitate multicohort studies.

**Methods:**

SONAR used semantic learning from variable descriptions and distribution learning from study participant data. Our method learned an embedding vector for each variable and used pairwise cosine similarity to score the similarity between variables. This approach was built off 3 National Institutes of Health cohorts, including the Cardiovascular Health Study, the Multi-Ethnic Study of Atherosclerosis, and the Women’s Health Initiative. We also used gold standard labels to further refine the embeddings in a supervised manner.

**Results:**

The method was evaluated using manually curated gold standard labels from the 3 National Institutes of Health cohorts. We evaluated both the intracohort and intercohort variable harmonization performance. The supervised SONAR method outperformed existing benchmark methods for almost all intracohort and intercohort comparisons using area under the curve and top*-k* accuracy metrics. Notably, SONAR was able to significantly improve harmonization of concepts that were difficult for existing semantic methods to harmonize.

**Conclusions:**

SONAR achieves accurate variable harmonization within and between cohort studies by harnessing the complementary strengths of semantic learning and variable distribution learning.

## Introduction

Data harmonization, the process that ensures the compatibility of diverse datasets for their cogent integration, is an indispensable tool in today’s data-driven research environment [1-3]. The power of data harmonization lies in its capacity to enhance the statistical robustness of studies, thereby enabling the investigation of intricate research questions unattainable within a single dataset’s limits. This ability to pool data from existing sources expedites research processes, reduces associated costs, and accelerates the translation of knowledge into practical applications [4,5]. However, despite the advantages of pooling data, the path to effective data harmonization is laden with challenges [6-9]. The most pronounced among these is the discrepancies in how individual datasets document and measure similar concepts [10-12]. Even within datasets, documentation for analogous concepts is not consistent, thereby further complicating data integration.

Current data harmonization techniques mainly depend on manual curation [10,13,14]. In spite of its widespread use, manual curation is time-intensive, prone to human error, and often constrained in scope, focusing predominantly on a single disease or condition. These drawbacks limit the applicability and efficacy of manual curation in broader, more complex contexts and highlight the need for advanced harmonization methodologies [12,15-17]. Recently, there has been a shift towards automated techniques, like freely available mapping tools [18-20] and algorithms based on corpora or lexicons [21-23]. These tools aim to map terminologies across varied clinical domains. Yet, such methods might still necessitate significant domain expertise and depend on benchmark labels. Furthermore, many of these methods cater to only one kind of medical code, for instance, drug or lab codes.

Another promising approach for data harmonization is through semantic learning. In a study by Zhou et al [24], an automated harmonization algorithm was proposed to cotrain embeddings for electronic health record codes from multiple institutions by combining both electronic health record co-occurrence information and textual information from the code descriptions. As a technique that uses machine learning to infer meaning from data, semantic learning presents a promising avenue for enhancing data harmonization. However, semantic learning’s applicability is limited by its demand for extensive, high-quality training data, its sensitivity to noisy or unreliable data, and the complexity involved in manually crafting semantic features.

In this paper, we propose SONAR (Semantic and Distribution-Based Harmonization), an innovative data harmonization approach that synthesizes the strengths of semantic learning with patient data learning. Patient data offers an alternative, unexplored source of learning for data harmonization purposes. The patient-level values for each variable provide information about the underlying concept that a variable measures, separate from the textual information in variable descriptions. By harnessing the context comprehension and inferential power of semantic learning and augmenting it with the capacity of patient data learning to capture concept-specific trends and nuances, we propose a more robust and accurate data harmonization strategy. We demonstrate the implementation and advantages of the proposed approach through its application across 3 major National Institutes of Health cohort studies: the Multi-Ethnic Study of Atherosclerosis (MESA) [25], the Cardiovascular Health Study (CHS) [26], and the Women’s Health Initiative (WHI) [27]. Our aspiration is that the method proposed here will provide a valuable foundation for future studies aiming to tackle the multifaceted challenges of data harmonization between heterogeneous datasets.

## Methods

### Ethical Considerations

The data used in this study were obtained from 3 well-established cohort studies, namely CHS, MESA, and WHI. Ethics approval (IRB17-2059) was granted by the Institutional Review Board of the Harvard Faculty of Medicine. Institutional Review Board approval was secured for access to all studies’ retrospective data. De-identified data was accessed through a secure cloud storage platform. Participants were not compensated for the use of their data in this study.

### Data Sources

The CHS was a population-based longitudinal study initiated to determine the risk factors for the development and progression of clinically validated cardiovascular disease in adults aged 65 years and older. Beginning in 1989, the study enrolled 5888 participants from 4 US communities: Forsyth County, NC; Sacramento County, CA; Washington County, MD; and Pittsburgh, PA. The cohort consisted of two recruitment waves: the original cohort (1989-1990) and the African American cohort (1992-1993). Comprehensive baseline examinations were conducted, including medical history, physical examinations, laboratory tests, and others, with annual follow-ups to ascertain cardiovascular events [26].

The WHI was a long-term national health study that focused on strategies for preventing heart disease, breast and colorectal cancer, and osteoporotic fractures in postmenopausal women. Launched in 1991, the WHI involved multiple clinical trials and an observational study, enrolling a total of 161,808 women aged 50-79 years across 40 clinical centers throughout the United States. The participants were ethnically diverse, reflecting the demographic composition of the US population. Extensive data on lifestyle, health, and medical history were collected at baseline and at regular intervals throughout the study, creating a rich source of information for a variety of research endeavors [27].

The MESA study was a prospective cohort designed to delve into the prevalence and progression of subclinical cardiovascular disease among community-dwelling adults. MESA assessed a diverse, population-based sample of 6814 asymptomatic men and women aged between 45 and 84 years from 2000 to 2018. The participants were recruited from 6 field centers across the United States, including Wake Forest University, Columbia University, Johns Hopkins University, University of Minnesota, Northwestern University, and University of California – Los Angeles. The MESA cohort was made up of 38% White, 28% African-American, 22% Hispanic, and 12% Asian (primarily Chinese) individuals. Since its inception in July 2000, the study conducted 6 examinations, each occurring every 18 to 24 months [25].

### Data Extraction

The process of data extraction necessitated the gathering of documentation for variables within the CHS, MESA, and WHI studies. This information was procured from the Database of Genotypes and Phenotypes (dbGaP) [28]. We used dbGaP metadata to procure the following salient information for each variable within the study: (1) variable accession, (2) variable name, (3) variable description, and (4) dataset accession. While the variable name and variable description were not necessarily unique within or between studies, the variable accession was a unique identifier across all studies. We used the variable description strings as the semantic data in our model. From the raw variable description strings, we further extracted and removed the temporal period during which the variables were measured by parsing for key temporal terms, such as visit and exam. This data extraction process facilitated a comprehensive understanding of the variables’ conceptual characteristics, thereby providing the foundation for the subsequent data harmonization efforts.

We used the dataset accession and variable accession identifiers to access (1) variable metadata and (2) the patient-level data for the set of variables already extracted from dbGaP. To allow for relevant distribution comparisons between variables, we kept only continuous data by filtering variables using the continuous flag in the metadata.

### Data Preprocessing

Our study scope was primarily focused on the harmonization of continuous variables at the conceptual level. A “concept” in this context was defined as the underlying notion or theme that a variable represents, independent of the specific unit or time point of measurement. For example, a biomarker such as C-reactive protein, despite being reported in different units across different visits, was treated as having the same concept. Moreover, concepts were sometimes encoded in natural language or questionnaire form, rather than standard medical terms. We focused on conceptual level harmonization for several reasons. Researchers conducting multicohort studies are often interested in identifying all variables corresponding to a concept. Depending on the application, they might be interested in further refining this concept-level harmonization or also harmonizing the variable values across different units or collection time periods. Removing temporal information and units allowed us to focus on the essential meaning or theme underlying the variable, thereby facilitating the primary task of concept-level harmonization, which is manually challenging and resource intensive, paving the way for further data harmonization. Moreover, doing so enhanced comparability across studies, as variables with the same concept were treated as equivalent, irrespective of the units used. During the initial phase of data preprocessing, we streamlined variable descriptions by eliminating temporal information phrases. This practice not only simplified the descriptions but also augmented their comparability.

We also applied filters to variables according to their values. First, we removed variables with incomplete patient data. To preserve a significant portion of variables, we considered incomplete patient data at the subgroup level rather than the individual patient level. We defined patient subgroups using the anchor variables of age, race, and sex. Per the characteristics of the study populations and data availability, we defined 4 age buckets (≤59 years, 60 to 69 years, 70 to 79 years, and ≥80 years). For the categorical anchor variables, we used 2 predefined race categories (White and Black) and 2 predefined sex categories (female and male), yielding a total of 16 possible patient subgroups (calculated as 4×2×2). For each study, we removed variables that had no patient data for one or more subgroups, considering only patient subgroups that were represented in the cohort.

Second, we purged variables that had uniformly zero values across all patients. This removal was necessary as variables without variability do not offer predictive power and thus, contribute little to subsequent analyses. Subsequently, we identified variables within the same study with identical descriptions and treated them as a single entity. Rather than maintaining these as separate variables, we amalgamated their distribution vectors by computing their element-wise mean. This consolidation concurrently reduced redundancy and bolstered the statistical power and robustness of downstream analyses. The underlying principle driving these measures was the emphasis on the core conceptual content encapsulated within variables, a focus that lays the groundwork for a more efficient and meaningful process of data harmonization.

### Creation of Gold Standard Labels

To assess the harmonization accuracy of SONAR, we manually created a set of gold standard labels. The process began with the curation of a concept list, consisting of common diseases, laboratory results, and medications, consistent with the goal of harmonization at the conceptual level (details in Multimedia Appendix 1, Section 4). With the concept list in hand, each of the 3 independent reviewers assigned raw variables from all 3 studies to the corresponding concepts based on their descriptions. Not all variables had labels, since the curated concept list was not comprehensive of all underlying concepts present in the studies. To ensure consistency and accuracy, we adopted a consensus-based approach for handling any discrepancies among the reviewers. In cases of disagreements, the reviewers discussed their rationales for their assignments, and through a process of discussion, literature review, and majority vote, they reached a consensus on the appropriate concept assignment. This rigorously prepared set of annotations, backed by consensus, formed our gold standard labels. In particular, each pair of variables corresponding to the same underlying concept formed a gold standard pair (ie, a pair of variables that a harmonization algorithm should map to each other). Such pairs consisted of variables from two different datasets (intercohort) or the same dataset (intracohort), since multiple variables from a single dataset could correspond to the same underlying concept. These labels offered a reliable standard against which we could validate our semantic learning and patient data learning techniques.

### SONAR (Semantic and Distribution-Based Harmonization)

The proposed SONAR approach had 4 steps, including semantic learning, distribution learning, concatenation of the two learnings, and supervised training. Underpinning both semantic learning and distribution learning was the idea that variables with similar textual descriptions and patient-level value distributions were more likely to encode the same underlying concept.

#### Step 1: Semantic Learning

We combined two existing pretrained large language models (LLMs) for semantic learning, CODER (Crosslingual Knowledge-Infused Medical Term Embedding) [29] and SapBERT (Self-Alignment Pretraining for Biomedical Entity Representations) [30]. CODER, a semantic representation learning tool, is a type of pretrained language model using a contrastive learning framework [30,31]. It is particularly suited to the biomedical terms and descriptions found in clinical studies because it was trained on terms, concepts, and their relations in the Unified Medical Language System (UMLS) [32] knowledge graph. SapBERT is a pretrained, masked learning model also trained on synonyms in the UMLS knowledge graph. Both CODER and SapBERT create embedding vector representations from textual input, which were the variable description strings in our method. Combining CODER and SapBERT, which use different pretraining algorithms and training sets within the same knowledge graph, allowed us to increase the robustness of our semantic embeddings. The advantage of using these existing language models pretrained was a balance between saved training time and specificity to the domain and task. The output of this step was a CODER embedding vector (*VAR_coder*) and a SapBERT embedding vector (*VAR_sapbert*) for each variable. The goal of this process was to transform the variable descriptions into a uniform, computable format that captured their semantic essence.

#### Step 2: Distribution Learning

In order to conduct comparisons of patient-level values for pairs of variables, we constructed vectors encoding the distributions of variables. For each study, we aggregated patients into the previously defined 16 subgroups, using the anchor variables of age, race, and sex. These anchor variables were present in all studies and clinically relevant to most of the other study variables. It was possible and permissible that the number of anchor groups varied across different cohorts. For instance, in the WHI cohort that consisted only of women, the number of anchor groups was reduced to 8 (4×1×2). Then, we computed the subgroup quartiles (ie, the 25th, 50th, and 75th percentiles), thus yielding a numerical distribution vector for each variable (*VAR_dist*) of up to length 48 (16×3). This process was designed to capture the distribution characteristics of each variable within defined anchor groups, thereby adding a computationally efficient layer of contextual understanding to our harmonization strategy. Moreover, the quartile distribution encoding strategy allowed for greater flexibility in data handling by preserving patient confidentiality.

#### Step 3: Concatenation

This stage combined the insights gained from semantic learning and distribution learning. Specifically, for each variable, we concatenated its *VAR_coder*, *VAR_sapbert*, and *VAR_dist* vectors into a single *VAR_concat* vector. In order to ensure standardized comparisons with *VAR_concat* vectors of the same length, we kept only the variable distribution dimensions in *VAR_dist* corresponding to anchor groups present in both cohorts in each interdataset harmonization. We kept all available variable distribution dimensions for intradataset harmonization. Prior to concatenation, we also normalized the *VAR_coder*, *VAR_sapbert*, and *VAR_dist* vectors separately to ensure they operated on the same scale. In particular, we took the absolute magnitude of each element in a vector, selected the maximum value among these values, then divided the original vector by the maximum absolute value. After normalization in this manner, the elements in each vector were bound to the −1 to 1 range. This was crucial because it ensured that no vector’s magnitude dominated during the concatenation process, thereby preserving the integrity of the information they conveyed. We then concatenated the normalized *VAR_coder*, *VAR_sapbert*, and *VAR_dist* vectors, resulting in a vector for each variable that we denoted as *VAR_concat* with dimension *d*. This concatenated vector, containing both semantic and distribution information, formed the foundation of our automated harmonization strategy.

#### Step 4: Supervised Training

We further refined the SONAR method through supervised training of a d×d rotation matrix *M* through gradient descent of a loss function (details in Multimedia Appendix 1, Section 1). The supervised embeddings were then the cross product of the unsupervised SONAR embeddings and *M*. Overall, this 4-step process served to capture the nuances and complexities of variable-concept relationships in a computationally efficient and robust manner. The systematic integration of semantic learning with distribution learning offered an innovative approach to data harmonization, promising to enhance accuracy, efficiency, and overall applicability of harmonization strategies.

### Evaluation Metrics

We assessed the performance of the proposed SONAR approach both within individual cohorts (intracohort) and between different cohorts (intercohort). We considered the area under the curve (AUC) of the receiver operating characteristic curve as the overall metric for harmonization accuracy. Specifically, for each underlying concept, we first computed the cosine similarity of the embedding vectors for known concept pairs (true positives) and an equal number of randomly selected concept pairs (false positives), where the cosine similarity measured the cosine of the angle between 2 vectors in a multidimensional space, effectively quantifying how similar they are. The AUC was then calculated, summarizing the overall accuracy of our method across varying decision thresholds for a given underlying concept. The overall AUC was the average of the concept-level AUC values. To reduce the effect of outliers within the sampled negative pairs, we averaged the 3 overall AUC values calculated for 3 sets of sampled negative pairs.

We also evaluated the performance of SONAR on hard concepts, defined to be concepts for which the benchmark SapBERT AUC was below the threshold of 0.900. The hard AUC was the average of the concept-level AUC values for the hard concepts. This was an important metric for demonstrating the added value of distribution learning and more broadly the complementary effects of the two forms of learning.

To further scrutinize the performance of our method, we reported the top*-k* accuracy (acc@*k*) for mapping of codes from Cohort A to Cohort B, where the cohorts were identical for intradataset mapping. For variable a in Cohort A, we let $B_{a}$ be the set of variables within Cohort B with embeddings that had the largest cosine similarity with variable a, and we let $G_{a}$ be the set of variables within Cohort B that were in a positive gold standard pair with variable a. Then, the acc@*k* for an underlying concept for the mapping from Cohort A to Cohort B was the number of codes a such that $B_{a}\cap G_{a}\neq 0$ divided by the total number of gold standard variables in Cohort A corresponding to the underlying concept. The acc@*k* for the mapping from Cohort A to Cohort B was then the average of the acc*@k* across all underlying concepts. This prevented dominance by underlying concepts with many corresponding gold standard variables. To calculate the intracohort acc@*k*, we first computed the intracohort acc@*k* for each underlying concept by averaging the acc@*k* for that underlying concept across all 3 intracohort comparisons (intra-CHS, intra-MESA, and intra-WHI). Underlying concepts were only averaged over the comparisons for which they were relevant, so the intracohort acc@*k* for a concept that had gold standard pairs in only CHS and MESA was be the average of the CHS acc@*k* and the MESA acc@*k* for the concept in question. Then, the intracohort acc@*k* was the average of the acc@k across all underlying concepts. The intercohort acc@*k* was calculated similarly, except across the 6 intercohort mappings (CHS to MESA, CHS to WHI, MESA to CHS, MESA to WHI, WHI to CHS, WHI to MESA). Finally, the overall acc*@k* was calculated across the 9 total mappings for each underlying variable. We obtained acc@*k* values for *k* values of 1, 3, 5, 10, 20 in order to provide additional insight into SONAR’s effectiveness at capturing true positives at different thresholds. These rigorous evaluations allowed us to confidently assert the efficacy of our harmonization method in both intracohort and intercohort settings.

For the supervised portion of our method, we used 2-fold cross-validation. We pooled cosine similarity values from both rounds of training before AUC calculations and averaged the 2 resulting cosine similarity values for non–gold standard pairs before acc@*k* calculations. To provide a comprehensive understanding of our method’s performance, we compared the AUC and other accuracy metrics obtained by SONAR with those obtained when using semantic learning (BioBERT [Bidirectional Encoder Representations from Transformers for Biomedical Text Mining], CODER, SapBERT) or distribution learning alone. We also obtained metrics for the concatenated semantic portion of SONAR (ie, CODER concatenated with SapBERT) to further highlight the added value of distribution learning. This comparative approach allowed us to illuminate the relative strengths and contributions of the individual components of our method and the added value achieved by their combination.

## Results

### Data Extraction and Preprocessing

**Figure 1.**
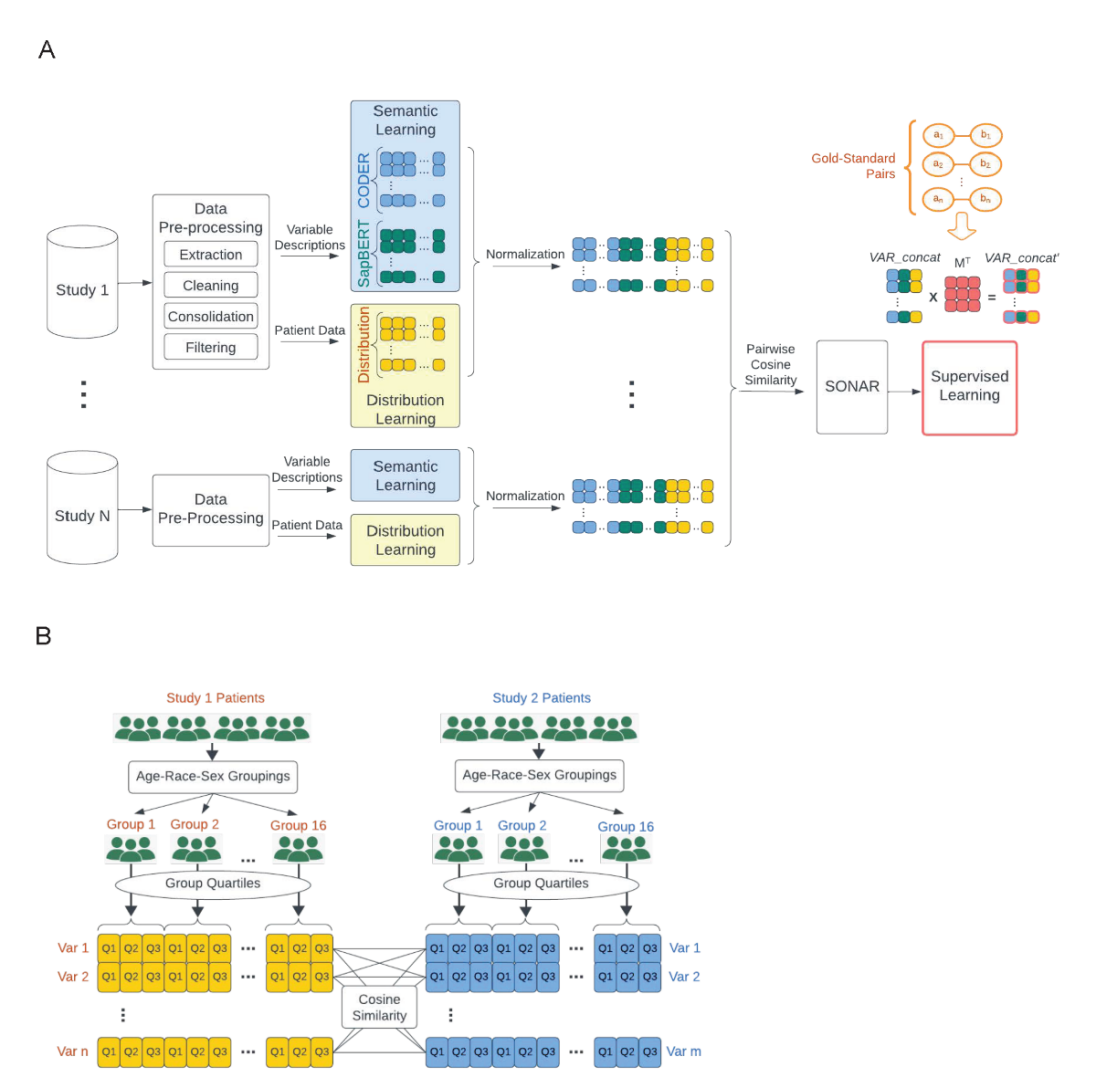
Workflow of semantic and distribution based harmonization. (A) Overall workflow of semantic and distribution based harmonization. (B) Workflow of the distribution learning step. CODER: Crosslingual Knowledge-Infused Medical Term Embedding; SapBERT: Self-Alignment Pretraining for Biomedical Entity Representations; SONAR: Semantic and Distribution-Based Harmonization. Var: variable.

The proposed SONAR approach had 4 steps, including semantic learning, distribution learning, concatenation of the two learnings, and supervised training (Figure 1). We extracted metadata and semantic data from the dbGaP for 14,717 CHS variables, 22,147 MESA variables, and 6207 WHI variables. Filtering for continuous, complete, and nonzero variables using metadata and patient-level data from Service WorkBench, as well as consolidating variables with identical semantic descriptions yielded 2076 CHS variables, 2525 MESA variables, and 1328 WHI variables. Patient data was available for 12 subgroups, 16 subgroups, and 6 subgroups for CHS, MESA, and WHI, respectively. This yielded distribution vectors of length 36, 48, and 18 for intra-CHS, intra-MESA, and intra-WHI harmonization, respectively. Based on overlapping patient subgroups between the cohorts, we used distribution vectors of length 36, 12, and 18 for intercohort CHS-MESA, CHS-WHI, and MESA-WHI harmonization, respectively.

### Gold Standard Labels

We identified a total of 123 concepts with continuous data, consisting of 112 laboratory test concepts, 6 disease concepts, and 5 medication concepts. A total of 531 variables across all cohorts were identified as gold standard representations of these concepts. These yielded 606, 318, and 89 gold standard concept pairs for intradataset harmonization evaluation within the CHS, MESA, and WHI, respectively. For interdataset harmonization evaluation, we had 352, 325, and 133 gold standard concept pairs for CHS-MESA, CHS-WHI, and MESA-WHI, respectively. Detailed numerical summaries of gold standard labels are provided in Table 1.

**Table 1.** Data preprocessing and gold standard labels.

Variables	Intracohort, n			Intercohort, n		
	CHS^a^	MESA^b^	WHI^c^	CHS-MESA	CHS-WHI	MESA-WHI
dbGaP^d^ variables	14,717	22,147	6207	36,864	51,581	28,354
Preprocessed variables	2076	2525	1328	4601	3404	3853
Distribution dimensions	36	48	18	36	12	18
Gold standard concepts	53	39	32	53	54	48
Gold standard variables	204	125	86	242	229	146
Gold standard pairs	606	318	89	352	325	133

a CHS: Cardiovascular Health Study.

b MESA: Multi-Ethnic Study of Atherosclerosis.

c WHI: Women’s Health Initiative.

d dbGaP: Database of Genotypes and Phenotypes.

### Intracohort Evaluation

**Figure 2.**
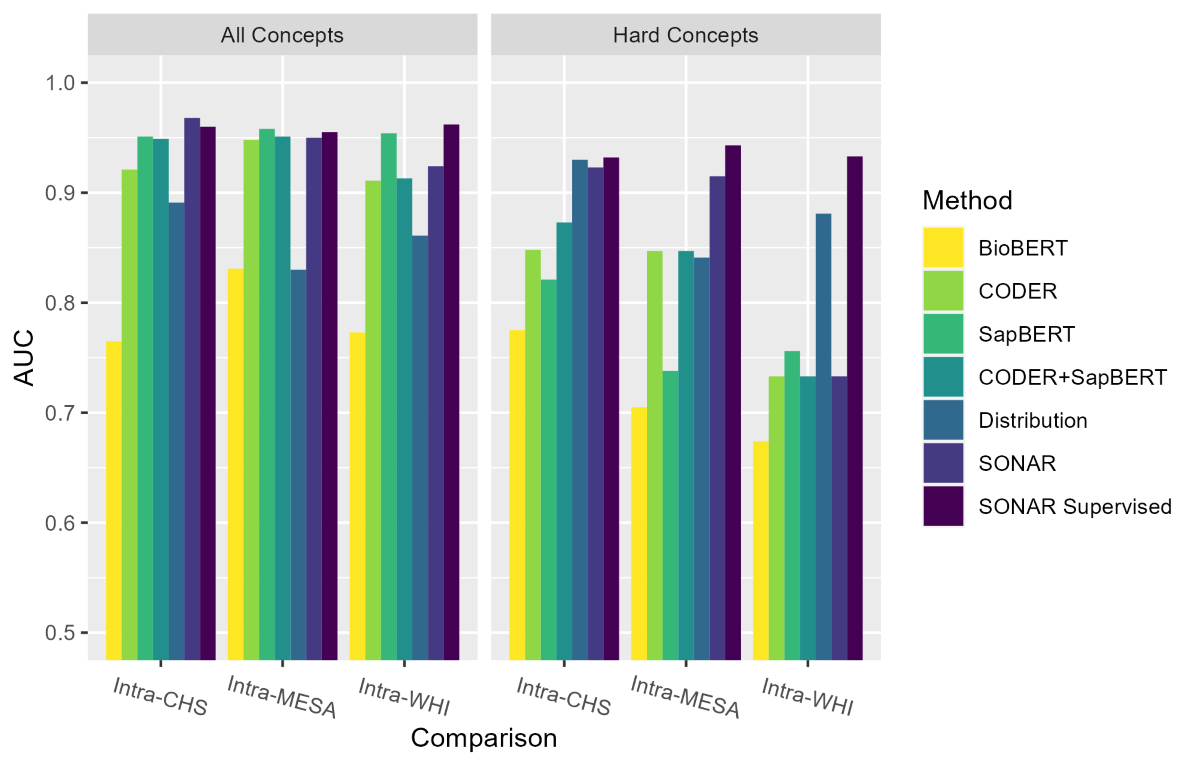
Comparison of areas under the curve for different methods in intracohort comparisons. AUC: area under the curve; BioBERT: Bidirectional Encoder Representations from Transformers for Biomedical Text Mining; CHS: Cardiovascular Health Study; CODER: Crosslingual Knowledge-Infused Medical Term Embedding; MESA: Multi-Ethnic Study of Atherosclerosis; SapBERT: Self-Alignment Pretraining for Biomedical Entity Representations; SONAR: Semantic and Distribution-Based Harmonization; WHI: Women’s Health Initiative.

**Figure 3.**
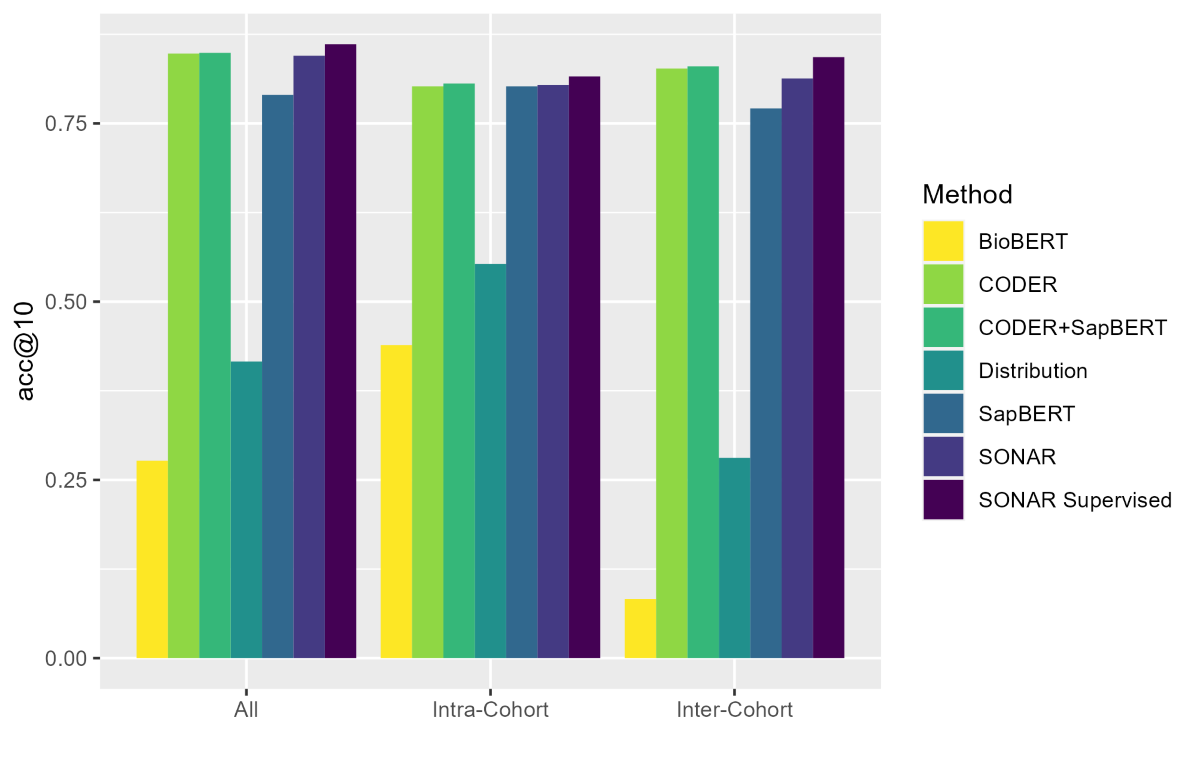
Comparison of top-10 sensitivities for different methods in both intracohort and intercohort comparisons. acc@10: top-10 accuracy; BioBERT: Bidirectional Encoder Representations from Transformers for Biomedical Text Mining; CODER: Crosslingual Knowledge-Infused Medical Term Embedding; SapBERT: Self-Alignment Pretraining for Biomedical Entity Representations; SONAR: Semantic and Distribution-Based Harmonization.

Supervised SONAR achieved a strong performance across all intracohort AUC (Figure 2 and Table S1 in Multimedia Appendix 1) and acc@*k* (Figure 3 and Table S2 in Multimedia Appendix 1) measures, exceeding or meeting all benchmark comparisons. The number of hard concepts for each intracomparison was 13 concepts, 5 concepts, and 5 concepts for intra-CHS, intra-MESA, and intra-WHI, respectively. It is notable that the distribution-only AUC was significantly higher than the semantic-only methods for the intra-CHS and intra-WHI hard concepts, illustrating the advantage of incorporating both semantic and distribution learning in SONAR. While the addition of supervised training only improved overall AUC performance for the intra-WHI comparison, it improved intracohort AUC performance on hard concepts for all 3 intracohort comparisons, exceeding all benchmark methods. The addition of supervised training also improved acc@*k* performance of SONAR across different values of *k*. Across intracohort evaluations, distribution learning provided a clear added value to semantic learning, in spite of a weaker distribution-only performance in comparison to the semantic components of SONAR (CODER only, SapBERT only, CODER + SapBERT). Moreover, there was not a single best semantic learning method between CODER and SapBERT using the various AUC and acc@*k* metrics, providing support for the use of both semantic learning methods in SONAR.

### Intercohort Evaluation

**Figure 4.**
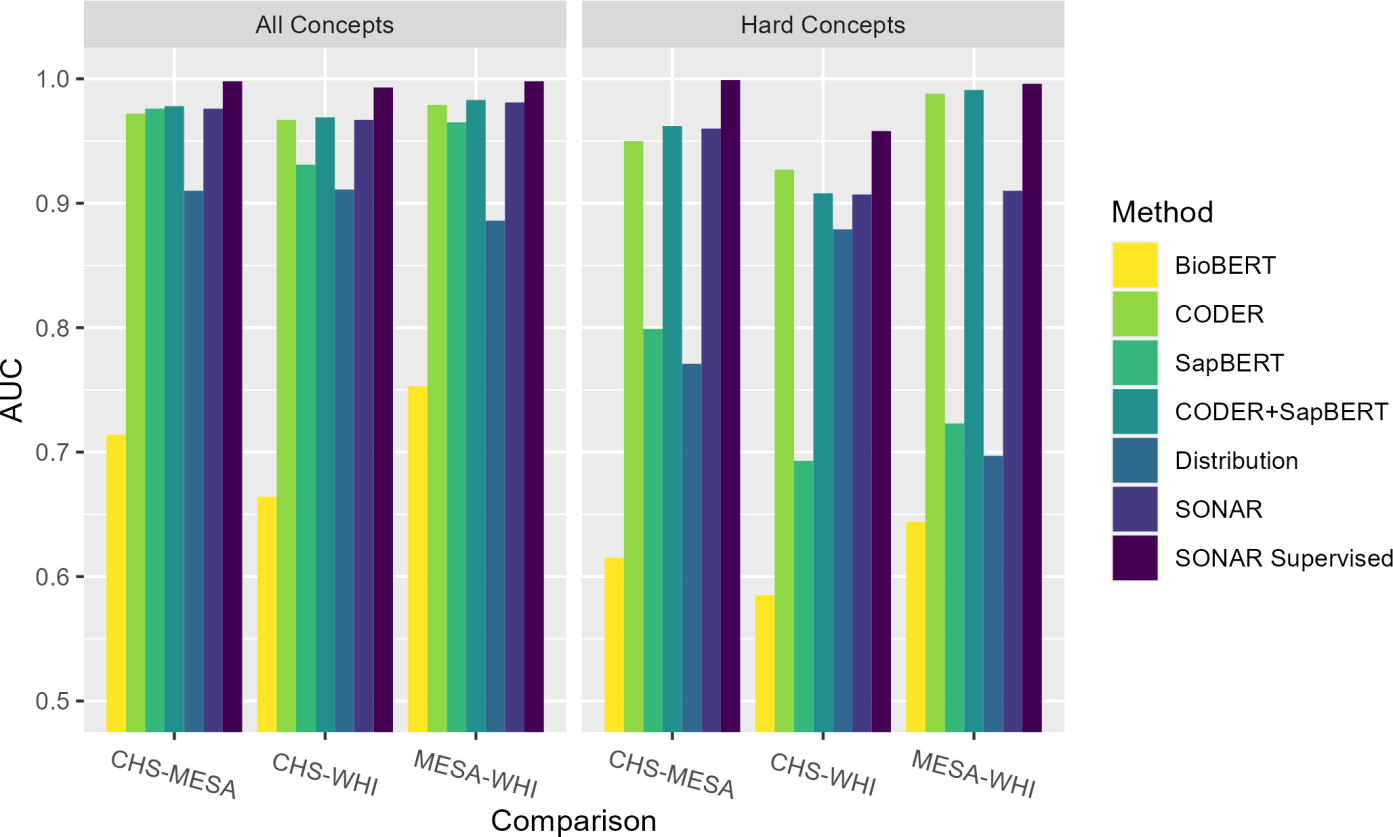
Comparison of areas under the curve for different methods in the intercohort comparisons. AUC: area under the curve; BioBERT: Bidirectional Encoder Representations from Transformers for Biomedical Text Mining; CHS: Cardiovascular Health Study; CODER: Crosslingual Knowledge-Infused Medical Term Embedding; MESA: Multi-Ethnic Study of Atherosclerosis; SapBERT (Self-Alignment Pretraining for Biomedical Entity Representations); SONAR: Semantic and Distribution-Based Harmonization; WHI: Women’s Health Initiative.

Supervised SONAR also achieved a consistently high performance in intercohort harmonization evaluation, exceeding or meeting all benchmark comparisons except for acc@3 and acc@20 (Figure 3). The number of hard concepts for each intracomparison was 4 concepts, 11 concepts, and 5 concepts for the CHS-MESA, CHS-WHI, and MESA-WHI comparisons, respectively. Similar to intracohort harmonization, supervised training improved AUC performance on hard concepts for all 3 comparisons, exceeding all benchmark methods. In contrast with intracohort harmonization, supervised training also improved AUC performance on all concepts to above 0.99 for all 3 intercohort comparisons (Figure 4). Notably, the CODER only and SapBERT only AUC values were higher for intercohort harmonization as compared to intracohort harmonization because identical variable descriptions were allowed for intercohort semantic learning.

## Discussion

The SONAR method provides a robust method for concept-level data harmonization across and within longitudinal cohort studies by efficiently constructing variable embeddings from longitudinal cohort study variable descriptions and data. We used a supervised algorithm to refine the concatenated embeddings built from normalized distribution and semantic vectors. When applied to harmonization within and between 3 National Institutes of Health cohort studies, SONAR achieved enhanced performance compared to benchmark methods, with notable improvements over semantic-only and distribution-only methods. These results demonstrate the effectiveness of learning from both semantic and patient-level data. Our method is able to conduct this learning with relatively low training costs by taking advantage of the one-time pretraining of biomedical entity representation–based language models using domain-specific UMLS terms.

There are some limitations to this study. We focused only on continuous variables with complete data, excluding categorical variables and variables with incomplete data. Future studies can expand variable distribution learning to categorical variables and develop methodologies for comparing distribution vectors of differing lengths in the case of variables with incomplete data, allowing for harmonization of a greater range of variables. Moreover, we focused on concept-level variable harmonization, which may be inappropriate for certain applications that require more granular harmonization, such as at the unit level or comparisons between different temporal periods. SONAR already drastically reduces the resources needed for concept-level harmonization, which is a crucial first step for more granular harmonization. Future studies could also automate the manual process of unit and temporal harmonization across variables corresponding to the same concept. Another direction for future research is automating the underlying concept identification process, perhaps by variable clustering using the newest generation of LLMs such as GPT-4. While powerful LLMs like GPT-4 could further improve the semantic learning aspect of our model, future research would need to adapt these generally trained models to the biomedical domain and control for the monetary costs associated with GPT-4 use. Additionally, although the current implementation of intercohort SONAR involves harmonization of 2 studies, it can be adapted to harmonize 3 or more studies.

SONAR paves the way for multicohort studies through high-quality and efficient variable harmonization. Harmonization at the concept-level is the crucial first step for researchers seeking to identify all variables corresponding to a disease, medication, or laboratory test of interest. Manual curation of or simple keyword searches for such variables are resource intensive and error-prone. The automation provided by SONAR is particularly helpful for harmonization of thousands of variables between large-scale cohort studies with heterogeneous variable encoding of underlying concepts. Multicohort studies that draw upon existing cohort studies are a resource-efficient method for studying risk factors associated with diseases and their pathogenesis. By effectively expanding the study population, multicohort studies also allow for greater statistical power and diversity in the study population, leading to greater generalizability of results and an enhanced ability to study health disparities. Beyond variable harmonization between cohorts, the variable embeddings generated through SONAR can be used for downstream analyses within multicohort studies, including for feature selection and the construction of knowledge graphs.

In conclusion, SONAR provides an approach for investigators to integrate semantic and patient data for multicohort variable harmonization. We demonstrate the robust added value of distribution learning when combined with existing semantic learning methods in variable mapping between cohorts. This innovation will facilitate and expedite multicohort studies by building upon existing data from decades-long cohort studies.

## Supplementary material

10.2196/54133 Multimedia Appendix 1 Revised supplementary material.

## Abbreviations

acc@k: top-*k* accuracy
AUC: area under the curve
BioBERT: Bidirectional Encoder Representations from Transformers for Biomedical Text Mining
CHS: Cardiovascular Health Study
CODER: Crosslingual Knowledge-Infused Medical Term Embedding
dbGaP: Database of Genotypes and Phenotypes
LLM: large language models
MESA: Multi-Ethnic Study of Atherosclerosis
SapBERT: Self-Alignment Pretraining for Biomedical Entity Representations
SONAR: Semantic and Distribution-Based Harmonization
UMLS: Unified Medical Language System
WHI: Women’s Health Initiative
