# Supplementary material for: Robust Automated Harmonization of Heterogeneous Data Through Ensemble Machine Learning: Algorithm Development and Validation Study
Source: JMIR Med Inform. 2025 Jan 22;13:e54133. doi: 10.2196/54133 (PMC11778729; doi:10.2196/54133)
Supplement: Multimedia Appendix 1 [file medinform-v13-e54133-s001.docx]

**Supplementary Materials**

Section 1. Supervised Loss Function:

Let $G=\{(a_{1},b_{1}), (a_{2},b_{2}), ..., (a_{g},b_{g})\}$ be the set of positive gold-standard pairs of variables between cohort A and cohort B. For intra-dataset comparisons, we consider cohort A and cohort B to be identical. Note that $a_{1}, ..., a_{g}$ and $b_{1}, ...., b_{g}$ need not be unique when more than two variables correspond to a single underlying concept. Thus, let $\{a'_{1}, ..., {a'}_{p}\}$ and $P=\{b'_{1}, ..., b'_{s}\}$ be the set of unique gold-standard variables among $a_{1}, ..., a_{g}$ and $b_{1}, ..., b_{g}$, respectively. Then let $P_{i}=\{b'_{i, 1}, ..., {b'}_{{i, m}_{i}}\}$ be the set of variables among ${b'}_{1}, ...., {b'}_{s}$ that form a positive gold-standard pair with $a'_{i}$, so that $P=\cup_{i=1}^{p} P_{i}$ and $g=\sum_{i=1}^{p} m_{i}$. Define $S_{ij}$ to be the cosine similarity between *VAR_concat*_$a'_{i}$ and *VAR_concat*_$b'_{i}\times M^{T}$. We then randomly sample $g$ “negative” pairs $G^{*}=\{({a^{*}}_{1},{b^{*}}_{1}), ({a^{*}}_{2},{b^{*}}_{2}), ..., ({a^{*}}_{g},{b^{*}}_{g})\}$ from the set of all pairs of variables between cohort A and cohort B. Using analogous notation, let $\{a^{*}'_{1}, ..., {a^{*}'}_{n}\}$ and and $N=\{b^{*}'_{1}, ..., b^{*}'_{t}\}$ be the set of unique variables among ${a^{*}}_{1}, ..., {a^{*}}_{g}$ and ${b^{*}}_{1}, ..., {b^{*}}_{g}$, respectively. Then let $N_{i}=\{b^{*}'_{i, 1}, ..., {b^{*}'}_{{i, q}_{i}}\}$ be the set of variables among ${b^{*}}_{1}, ...., {b^{*}}_{t}$ that form a negative pair with $a^{*}'_{i}$, so that $N=\cup_{i=1}^{p} N_{i}$ and $g=\sum_{i=1}^{n} q_{i}$. Define $R_{ij}$ to be the cosine similarity between *VAR_concat*_$a^{*}'_{i}$ and *VAR_concat*_$b^{*}'_{i}\times M^{T}$. We define the following contrastive learning objective function:

$$L(M) = \frac{1}{\alpha}\sum_{i=1}^{p} log(1+\sum_{j=1}^{m_{i}} exp(-\alpha(S_{ij}-\lambda))) + \frac{1}{\beta}\sum_{i=1}^{n} log(1+\sum_{j=1}^{q_{i}} exp(\beta(R_{ij}-\lambda)))$$

Optimization of this non-convex loss function involves the additional hyperparameters of step size, maximum steps, and $\varepsilon$. We initialize the rotation matrix $M_{0}$ with a diagonal matrix and update $M_{t+1}=M_{t}-\gamma\cdot\nabla L(M_{t})$ through gradient descent. Gradient descent continues until at least one of the two following stopping conditions is met: (1) the maximum number of gradient descent steps is reached and (2) the norm of the gradient matrix is less than $\varepsilon$. The trained embeddings are calculated using the final rotation matrix $M_{t}$ as *VAR_concat*_$b\times M^{T}$ for all variables in Cohort B. The hyperparameters $\alpha$ and $\beta$ can be chosen according to the relative weight given to positive pairs and negative pairs, respectively. When using randomly sampled pairs as negative pairs, $\alpha$ should be smaller than $\beta$ so as to upweight the positive gold-standard pairs. The hyperparameter $\lambda$ can be tuned to the target cosine similarity cutoff between positive and negative pairs. For our study, we set the hyperparameters to be $\alpha=2, \beta=50, \gamma=0.001, \varepsilon=0.01, \lambda=0.1$, and the maximum number of steps to be 200.

Note that the loss function, which is nonnegative, is minimized as the cosine similarity between positive pairs exceeds $\lambda$ and the cosine similarity between sampled negative pairs decreases below $\lambda$. Moreover, the structure of the loss function prevents overweighting of variables that are present in many gold-standard pairs.

Section 2. Supplementary Table 1: Comparison of AUCs for different methods both intra- and inter- cohorts.

|  | Intra-Cohort | | | | | | Inter-Cohort | | | | | |
| --- | --- | --- | --- | --- | --- | --- | --- | --- | --- | --- | --- | --- |
| Method | CHS | | MESA | | WHI | | CHS-MESA | | CHS-WHI | | MESA-WHI | |
| Concepts | All | Hard | All | Hard | All | Hard | All | Hard | All | Hard | All | Hard |
| Distribution only | 0.891 | 0.930 | 0.830 | 0.841 | 0.861 | 0.881 | 0.910 | 0.771 | 0.911 | 0.879 | 0.886 | 0.697 |
| BioBERT only | 0.765 | 0.775 | 0.831 | 0.705 | 0.773 | 0.674 | 0.714 | 0.615 | 0.664 | 0.585 | 0.753 | 0.644 |
| CODER only | 0.921 | 0.848 | 0.948 | 0.847 | 0.911 | 0.733 | 0.972 | 0.950 | 0.967 | 0.927 | 0.979 | 0.988 |
| SapBERT only | 0.951 | 0.821 | **0.958** | 0.738 | 0.954 | 0.756 | 0.976 | 0.799 | 0.931 | 0.693 | 0.965 | 0.723 |
| CODER + SapBERT | 0.949 | 0.873 | 0.951 | 0.847 | 0.913 | 0.733 | 0.978 | 0.962 | 0.969 | 0.908 | 0.983 | 0.991 |
| SONAR | **0.968** | 0.923 | 0.950 | 0.915 | 0.924 | 0.733 | 0.976 | 0.960 | 0.967 | 0.907 | 0.981 | 0.910 |
| SONAR supervised | 0.960 | **0.932** | 0.955 | **0.943** | **0.962** | **0.933** | **0.998** | **0.999** | **0.993** | **0.958** | **0.998** | **0.996** |

Section 3. Supplementary Table 2: Comparison of top-1, 3, 5, 10 and 20 sensitivities for different methods both intra- and inter- cohorts.

|  | Method | Top 1 | Top 3 | Top 5 | Top 10 | Top 20 |
| --- | --- | --- | --- | --- | --- | --- |
| All | Distribution only | 0.232 | 0.318 | 0.367 | 0.416 | 0.470 |
|  | BioBERT only | 0.204 | 0.242 | 0.246 | 0.277 | 0.305 |
|  | CODER only | 0.628 | 0.767 | 0.801 | 0.848 | 0.896 |
|  | SapBERT only | 0.616 | 0.683 | 0.741 | 0.790 | 0.821 |
|  | CODER + SapBERT | 0.639 | **0.771** | 0.803 | 0.849 | **0.909** |
|  | SONAR | 0.628 | 0.757 | 0.802 | 0.845 | 0.897 |
|  | SONAR supervised | **0.648** | 0.767 | **0.804** | **0.861** | 0.903 |
| Intra-Cohort | Distribution only | 0.368 | 0.454 | 0.509 | 0.553 | 0.601 |
|  | BioBERT only | 0.313 | 0.393 | 0.399 | 0.439 | 0.466 |
|  | CODER only | 0.539 | 0.687 | 0.733 | 0.802 | 0.868 |
|  | SapBERT only | 0.606 | 0.675 | **0.748** | 0.802 | 0.830 |
|  | CODER + SapBERT | 0.545 | 0.695 | 0.733 | 0.806 | 0.881 |
|  | SONAR | 0.544 | 0.688 | 0.746 | 0.804 | 0.860 |
|  | SONAR supervised | 0.552 | 0.708 | **0.748** | **0.816** | **0.888** |
| Inter-Cohort | Distribution only | 0.115 | 0.193 | 0.222 | 0.281 | 0.342 |
|  | BioBERT only | 0.047 | 0.057 | 0.062 | 0.083 | 0.115 |
|  | CODER only | 0.622 | 0.766 | 0.786 | 0.827 | 0.880 |
|  | SapBERT only | 0.596 | 0.672 | 0.714 | 0.771 | 0.799 |
|  | CODER + SapBERT | 0.636 | **0.767** | 0.791 | 0.830 | **0.893** |
|  | SONAR | 0.624 | 0.743 | 0.771 | 0.813 | 0.874 |
|  | SONAR supervised | **0.662** | 0.761 | **0.804** | **0.843** | 0.876 |

Section 4: Concept curation process

The concept curation process aimed to identify common diseases, laboratory results, and medications that frequently emerged in the scope of our chosen cohort studies. To ensure a wide range of concepts and generalizability of the concept list to other cohort studies, we began with a list of PheCodes (<https://phewascatalog.org/phecodes>) at the single decimal hierarchical level. We eliminated concepts that did not appear in our chosen cohort studies through keyword searches of the concepts and their common synonyms. Curation then involved a careful review of literature and existing databases, followed by a consensus-driven process among the research team to define and list these concepts. This curated list formed the basis for the subsequent annotation task.
